# Hotspots for Initiation of Meiotic Recombination

**DOI:** 10.3389/fgene.2018.00521

**Published:** 2018-11-05

**Authors:** Andrew J. Tock, Ian R. Henderson

**Affiliations:** Department of Plant Sciences, University of Cambridge, Cambridge, United Kingdom

**Keywords:** meiosis, recombination, DSB, crossover, hotspot, chromatin, nucleosomes, epigenetics

## Abstract

Homologous chromosomes must pair and recombine to ensure faithful chromosome segregation during meiosis, a specialized type of cell division that occurs in sexually reproducing eukaryotes. Meiotic recombination initiates by programmed induction of DNA double-strand breaks (DSBs) by the conserved type II topoisomerase-like enzyme SPO11. A subset of meiotic DSBs are resolved as crossovers, whereby reciprocal exchange of DNA occurs between homologous chromosomes. Importantly, DSBs are non-randomly distributed along eukaryotic chromosomes, forming preferentially in permissive regions known as hotspots. In many species, including plants, DSB hotspots are located within nucleosome-depleted regions. DSB localization is governed by interconnected factors, including *cis*-regulatory elements, transcription factor binding, and chromatin accessibility, as well as by higher-order chromosome architecture. The spatiotemporal control of DSB formation occurs within a specialized chromosomal structure characterized by sister chromatids organized into linear arrays of chromatin loops that are anchored to a proteinaceous axis. Although SPO11 and its partner proteins required for DSB formation are bound to the axis, DSBs occur preferentially within the chromatin loops, which supports the “tethered-loop/axis model” for meiotic recombination. In this mini review, we discuss insights gained from recent efforts to define and profile DSB hotspots at high resolution in eukaryotic genomes. These advances are deepening our understanding of how meiotic recombination shapes genetic diversity and genome evolution in diverse species.

## 1. Introduction

Meiosis is a specialized cell division program that is essential for sexual reproduction in eukaryotes. During this program, replication of chromosomal DNA to form sister chromatids is followed by two rounds of cell division. Maternal and paternal chromosomes (homologs) segregate at the first division and sister chromatids segregate at the second division. Chromosome number is thereby halved and, in diploid organisms, meiosis culminates in the production of haploid progeny cells (gametes). Chromosome segregation during meiosis is imperative for the continuation of the species, as it enables the formation of a zygote that inherits the full chromosome complement in the next generation by fusion of a male and a female gamete (Villeneuve and Hillers, 2001). DNA double-strand breaks (DSBs) occur at many genomic loci during early prophase I to initiate meiotic recombination, whereby pairing of and reciprocal DNA exchange (crossover) between homologous chromosomes promote their balanced segregation and genetic diversity (De Massy, 2013; Keeney et al., 2014). Meiotic DSBs form preferentially in permissive regions known as hotspots, giving rise to non-random DSB and crossover distributions that influence patterns of genetic linkage and genome evolution in eukaryotes (Baudat et al., 2013; Cooper et al., 2016). The genome-wide distribution and the resolution of a subset of DSBs as crossovers have immediate impacts on haplotype configurations in the recombinant gametes, as well as far-reaching, population-level consequences for locus-specific rates of genetic change over evolutionary time (Cooper et al., 2016).

Meiotic DSBs are catalyzed by SPO11 dimers in a type II topoisomerase-like reaction in which one SPO11 molecule becomes covalently bound to each 5′ end of the cleaved DNA (Bergerat et al., 1997; Keeney et al., 1997). To enable DSB repair as a crossover or a non-crossover, the two SPO11–oligonucleotide complexes are endonucleolytically released (Neale et al., 2005) and 5′–3′ resection exposes a 3′-overhanging, single-stranded DNA (ssDNA) tail at each end of the DSB (Cao et al., 1990; Sun et al., 1991; Zakharyevich et al., 2010). The meiotic recombinases DMC1 and RAD51 bind these ssDNA tails and promote the search for a homologous chromosome to provide a template for DNA repair (Bishop et al., 1992; Shinohara et al., 1992; Cloud et al., 2012). Following invasion of the homolog, strand-exchange intermediates can be processed via different DNA repair pathways to produce non-crossovers or crossovers (Hunter, 2015). Most non-crossovers are products of synthesis-dependent strand annealing (SDSA), whereby the homolog-invading DSB end initiates DNA synthesis and is subsequently displaced and annealed to the other end of the DSB (Pâques and Haber, 1999; McMahill et al., 2007). Non-crossovers can also result from dissolution of double Holliday junction joint molecules (dHJ-JMs) by combined helicase and topoisomerase activities (Cejka et al., 2010), or from unidirectional endonuclease cleavage of dHJ-JMs (Szostak et al., 1983; De Muyt et al., 2012). However, most if not all stable dHJ-JMs are resolved as crossovers during meiosis (Allers and Lichten, 2001; Hunter and Kleckner, 2001; Hunter, 2015).

Efforts to generate genome-wide, nucleotide-resolution maps of eukaryotic DSB landscapes have recently intensified with the advent of techniques to immunoprecipitate SPO11 and end-label, purify and sequence SPO11-bound oligonucleotides, which are a byproduct of DSB formation (Pan et al., 2011). SPO11-oligo mapping by these means has been applied in several budding yeast species, fission yeast, mouse and *Arabidopsis thaliana* (Pan et al., 2011; Fowler et al., 2014; Thacker et al., 2014; Lam and Keeney, 2015; Zhu and Keeney, 2015; Lange et al., 2016; Mohibullah and Keeney, 2017; Choi et al., 2018; Underwood et al., 2018). Parallel advances have been achieved by exploiting meiotic ssDNA formed at resected DSB ends for high-resolution mapping of recombination initiation sites in budding yeast, maize, mouse and human genomes (Blitzblau et al., 2007; Buhler et al., 2007; Borde et al., 2009; Smagulova et al., 2011; Brick et al., 2012; Khil et al., 2012; Pratto et al., 2014; Lange et al., 2016; He et al., 2017). Single-stranded DNA sequencing (SSDS) utilizes chromatin immunoprecipitation followed by high-throughput sequencing (ChIP-seq) of ssDNA bound by the strand-exchange proteins DMC1 and RAD51, thereby capturing chromosome fragments that are immediately adjacent to DSB sites (Smagulova et al., 2011; Khil et al., 2012). We discuss insights gained from these high-resolution physical maps of meiotic DSB landscapes, highlighting the genetic and epigenetic properties of hotspots for recombination initiation in different eukaryotic species.

## 2. Defining meiotic DSB hotspots

Maps describing recombination initiation profiles at nucleotide resolution have revealed that hotspots constitute one of several levels of DSB patterning (Cooper et al., 2016). The genome-wide DSB landscape is most accurately characterized as a continuous probability distribution, where DSB hotspots are defined as genomic loci with high local likelihoods of DNA cleavage by SPO11 (Pan et al., 2011). Most if not all genomic loci are sites of potential cleavege and many DSBs form in regions not defined as hotspots (Pan et al., 2011). The limits of DSB detection are determined by methodological constraints associated with quantifying the signal to noise ratio at each locus, which vary between organisms due to differences in both their underlying biology and the methodologies adopted. Thus, while the “hotspot” concept is useful for annotating preferred sites and identifying the possible determinants of recombination initiation activity, comparisons of quantitative measurements taken across species and methodologies should be considered with caution. In yeast species (*Saccharomyces cerevisiae, S. kudriavzevii, S. mikatae, S. paradoxus*, and *Schizosaccharomyces pombe*) and mouse, DSB hotspots have been defined as loci meeting or exceeding given thresholds for Spo11-oligo density and physical size (Pan et al., 2011; Fowler et al., 2014; Lam and Keeney, 2015; Zhu and Keeney, 2015; Lange et al., 2016; Mohibullah and Keeney, 2017). The accuracy of this method for DSB hotspot definition has been validated by comparing the spatial patterning of yeast Spo11-oligo maps with DSBs assayed directly by Southern blotting of genomic DNA from yeast meiocytes (Pan et al., 2011).

A false discovery rate (FDR)-based peak-calling approach (Feng et al., 2011) was adopted to identify loci in the *Arabidopsis* genome with significantly higher-than-expected SPO11-1-oligo enrichment, using the binomial distribution to model enrichment relative to a control library derived from genomic DNA (Choi et al., 2018). Peaks identified in replicate SPO11-1-oligo libraries were ranked by their −log_10_-transformed FDR values and *Arabidopsis* DSB hotspots were defined as peaks with consistent rankings between replicates (i.e., peaks with irreproducible discovery rates [IDR] <0.05) (Li et al., 2011; Choi et al., 2018). Similar peak-calling approaches have been employed to define DSB hotspots derived from SSDS in maize, mouse and human genomes (Smagulova et al., 2011, 2016; Brick et al., 2012; Khil et al., 2012; Pratto et al., 2014; He et al., 2017), with confirmation of a sample of hotspots by qPCR or direct physical detection methods (Smagulova et al., 2011). Positive genome-wide associations between DSB maps and genetic or crossover maps provide further validation of these recombination initiation site mapping approaches (Smagulova et al., 2011; Pratto et al., 2014; Choi et al., 2018). Mouse DSB maps obtained by SPO11-oligo mapping and SSDS also show a high level of agreement (Lange et al., 2016).

Hotspot density in fission yeast is substantially lower than in budding yeast genomes (one hotspot per 20.9 kb compared with one hotspot per ~3 kb, respectively; Table 1), consistent with substantially longer chromosomes and lower recombination frequencies in fission yeast (Pan et al., 2011; Fowler et al., 2014; Thacker et al., 2014; Lam and Keeney, 2015; Zhu and Keeney, 2015; Mohibullah and Keeney, 2017). Another important difference to consider is the absence of crossover interference in fission yeast (Munz, 1994). It is possible that DSB formation in fission yeast is restricted by competition between potential DSB sites for a more limited pool of recombination-promoting factors, thereby obviating the requirement for a downstream mechanism such as crossover interference to regulate the spacing of recombination events (Cooper et al., 2016). A more conservative approach to DSB hotspot definition was applied in *Arabidopsis*, based on the identification of reproducible SPO11-1-oligo peaks across biological replicates (Choi et al., 2018). This method is useful for minimizing the occurrence of false positives in peak sets, but likely underestimates the number of hotspots in the *Arabidopsis* genome, suggesting that hotspot density lies somewhere between those of budding yeast and fission yeast (Table 1). In mouse genomes, comparable hotspot numbers and densities were obtained by peak calling using SSDS data (Brick et al., 2012; Khil et al., 2012) and by enrichment thresholding using SPO11-oligo data (Lange et al., 2016), although SSDS-derived hotspots are wider on average (2-3.4 kb vs. 281 bp, respectively; Table 1), consistent with the action of resection. While mouse and human genomes are of similar size, hotspot numbers and densities in humans are more than double those in mice (Table 1) (Pratto et al., 2014). This is consistent with a more than doubled genome-wide average crossover frequency in human (1.20 cM/Mb) compared with mouse (0.528 cM/Mb) (Jensen-Seaman et al., 2004).

**Table 1:** Meiotic DNA double-strand break (DSB) hotspots identified in eukaryotes by SPO11-oligo mapping or single-stranded DNA sequencing (SSDS).

**Species (strain)**	**Genome size (Mb)**	**DSB hotspots**	Hotspot density (kb)^*^	**Average width (kb)**	**Method**	**Hotspot detection**	**Study**
*S. cerevisiae* (SK1)	12.123	3,604–4,099	2.958–3.364	0.248–0.264	Spo11-oligos	Enrichment threshold	Pan et al., 2011
							Thacker et al., 2014
							Zhu and Keeney, 2015
							Mohibullah and Keeney, 2017
*S. cerevisiae* (YPS128)	12.123	4,177	2.902	0.265	Spo11-oligos	Enrichment threshold	Lam and Keeney, 2015
*S. cerevisiae* (UW)^§^	12.123	3,881	3.124	0.256	Spo11-oligos	Enrichment threshold	Lam and Keeney, 2015
*S. kudriavzevii* (ZP591)	10.055	3,976	2.529	0.280	Spo11-oligos	Enrichment threshold	Lam and Keeney, 2015
*S. mikatae* (IFO1815)	11.121	3,829	2.904	0.269	Spo11-oligos	Enrichment threshold	Lam and Keeney, 2015
*S. paradoxus* (YPS138)	11.906	3,833	3.106	0.279	Spo11-oligos	Enrichment threshold	Lam and Keeney, 2015
*S. pombe* (GP6232)	12.608	603	20.909	1.400	Rec12-oligos	Enrichment threshold	Fowler et al., 2014
*A. thaliana* (Col-0)	119.668	5,914	20.235	0.823	SPO11-1-oligos	Peak calling	Choi et al., 2018
*Z. mays*	2,135.083	3,126	683.008	1.200	SSDS	Peak calling	He et al., 2017
*M. musculus* (F_1_)^†^	2,730.872	9,874–15,677	174.196–276.572	~2.000–3.400	SSDS	Peak calling	Smagulova et al., 2011
							Khil et al., 2012
							Brick et al., 2012
*M. musculus* (9R)	2,730.872	14,869	183.662	~2.000	SSDS	Peak calling	Brick et al., 2012
*M. musculus* (13R)	2,730.872	15,481	176.402	~2.000	SSDS	Peak calling	Brick et al., 2012
*M. musculus* (B6)	2,730.872	18,313	149.122	~2.000	SSDS	Peak calling	Brick et al., 2012
*M. musculus* (B6)	2,730.872	13,960	195.621	0.281	SPO11-oligos	Enrichment threshold	Lange et al., 2016
*H. sapiens*	3,096.650	38,946	79.511	1.500	SSDS	Peak calling	Pratto et al., 2014

§ UWOPS03-461.4;

† 9R × 13R F_1_ hybrids;

* *One DSB hotspot per indicated kilobase pairs*.

## 3. Chromatin shapes the meiotic DSB landscape

### 3.1. Nucleosome occupancy

DSB hotspot designation is controlled at multiple levels, with a hierarchy of “gatekeeper” factors acting in concert to determine the degree to which chromosome regions—at fine and broad scales—are conducive to DSB formation (Pan et al., 2011; De Massy, 2013; Cooper et al., 2016; Lange et al., 2016). Different strategies and mechanisms for the spatial regulation of DSB formation have evolved in different species, although commonalities exist (De Massy, 2013).

**Figure 1:**
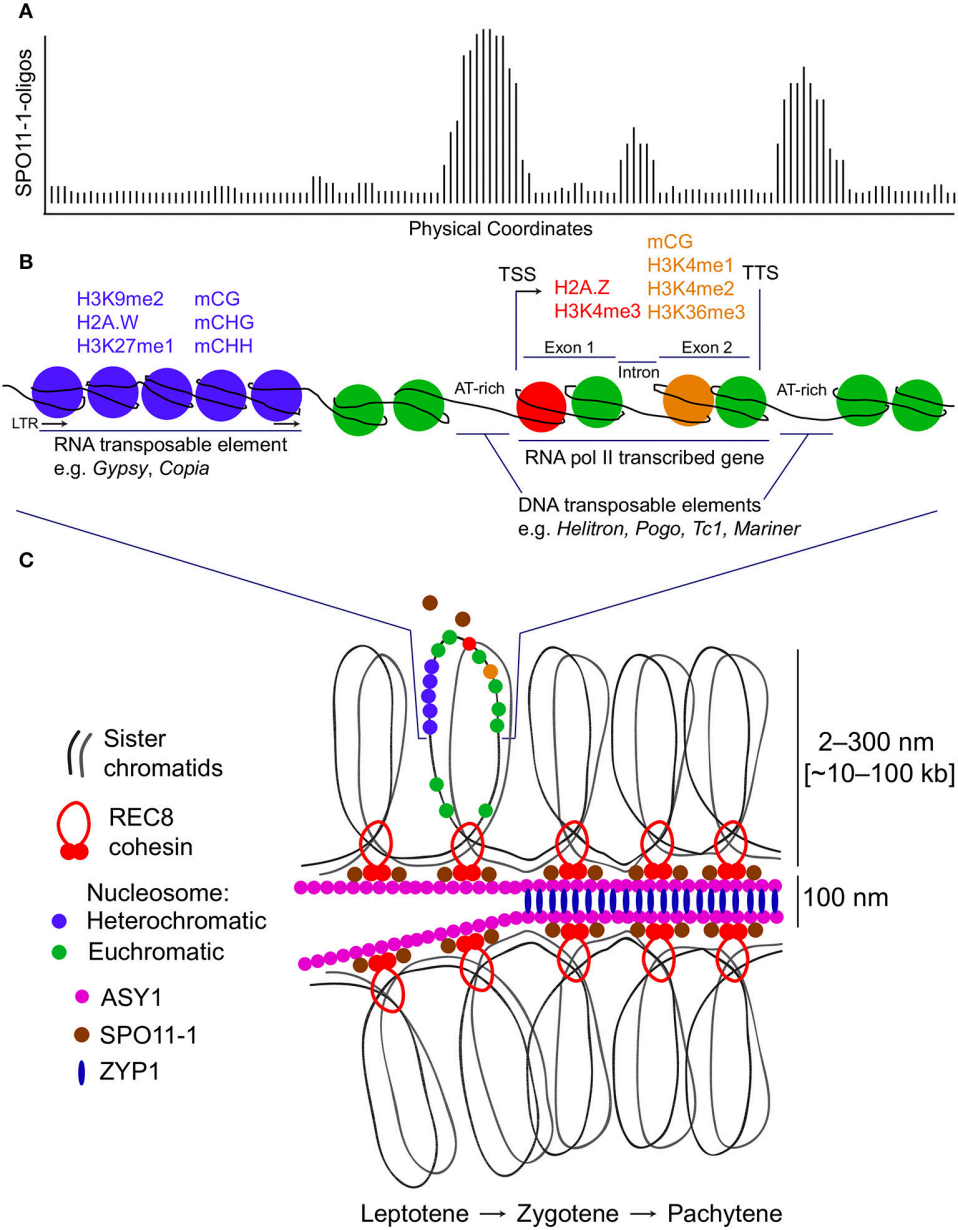
Meiotic DNA double-strand break hotspots, chromatin and chromosome architecture in plants. **(A)** A representative histogram showing relative levels of meiotic DNA double-strand breaks (DSBs) generated by SPO11-1. Physical coordinates along a hypothetical locus are represented on the x-axis and DSB signal intensity derived from SPO11-1-oligo mapping is indicated on the y-axis. The depicted hypothetical DSB topology maps on to the chromatin diagram in **(B)**. **(B)** A representative chromosomal region is shown based on data from *Arabidopsis thaliana*. This region contains an LTR retrotransposon which has heterochromatic modifications (blue), including H3K9me2, H2A.W, H3K27me1, and DNA methylation in CG, CHG, and CHH sequence contexts. Adjacent is an RNA polymerase II transcribed gene with transcriptional start site (TSS) and termination site (TTS) indicated. The 5′ nucleosome within the gene contains H2A.Z and is H3K4me3 modified (red). Within the transcribed region, nucleosomes located toward the 3′ end are H3K4me1, H3K4me2, and H3K36me3 modified, and DNA methylated in the CG sequence context (orange). The regions of highest meiotic DSB formation correspond to gene promoter, terminator and intron regions, which tend to be AT-rich, nucleosome-depleted and contain insertions of DNA transposable elements. **(C)** The chromatin region shown in **(B)** is represented in the context of the tethered-loop/axis model for meiotic chromosomes. SPO11-1 is represented as both a freely diffusing pool and an axis/cohesin-associated pool. Paired sister chromatids organize as a linear loop array on an axial polymer, which includes ASY1. As meiosis progresses from leptotene to zygotene to pachytene, the homologs become synapsed at a distance of ~100 nm, with ZYP1 installed as transverse filaments of the synaptonemal complex. During this process, DSBs can undergo repair using a homologous chromosome, resulting in a crossover (not shown).

Genome-wide DSB maps for *Saccharomyces* species, *Arabidopsis* and maize have revealed that hotspots frequently occur within nucleosome-depleted regions (NDRs) in gene promoters (Figure 1), indicating that local chromatin accessibility contributes to DSB formation in these eukaryotes (Pan et al., 2011; Lam and Keeney, 2015; He et al., 2017; Choi et al., 2018). AT-sequence richness at budding yeast, *Arabidopsis* and tomato recombination hotspots is thought to exclude nucleosomes and thereby permit increased SPO11 recruitment to chromatin, promoting DNA cleavage and ultimately crossover formation (Pan et al., 2011; Choi et al., 2013, 2018; Wijnker et al., 2013; Shilo et al., 2015; Demirci et al., 2018). Indeed, elevated crossover recombination within gene promoters is conserved in several eukaryotes, including plants, canids and birds (Auton et al., 2013; Choi et al., 2013; Wijnker et al., 2013; Singhal et al., 2015; Demirci et al., 2017). The +1 nucleosomes of *Arabidopsis* genes whose promoters exhibit the highest crossover frequencies show greater deposition of the histone variant H2A.Z and enrichment of trimethylated lysine 4 on histone H3 (H3K4me3) (Choi et al., 2013), which are key determinants of transcriptional regulation (Deal and Henikoff, 2011; Coleman-Derr and Zilberman, 2012; Sura et al., 2017). Reduction of crossover frequency at crossover hotspots in the *arp6* H2A.Z-deposition mutant confirmed a role for this histone variant in promoting recombination (Choi et al., 2013). Additionally, fewer RAD51 and DMC1 foci were observed in *arp6* mutants, indicating that the recombination-promoting role of H2A.Z may include control of DSB numbers and localization (Choi et al., 2013). H2A.Z may also indirectly promote recombination by maintaining the boundaries of NDRs at which DSBs form.

*Arabidopsis* SPO11-1-oligos also cluster in NDRs immediately downstream of transcription termination sites and in nucleosome-depleted introns (Figures 1A,B), suggesting a role for gene architecture in determining DSB positioning (Choi et al., 2018). Similarly, avian recombination rates toward the 5′ and 3′ ends of gene bodies (in both exons and introns) are elevated compared with more central regions (Singhal et al., 2015). In isolation, however, the presence of euchromatin does not adequately account for DSB hotspot locations, as NDRs immediately downstream of gene stop codons are not enriched for Spo11-oligos in budding yeast unless they overlap a promoter NDR (Pan et al., 2011). Furthermore, fission yeast DSBs do not form preferentially in NDRs, but rather at the boundaries between NDRs and well-positioned nucleosomes as inferred from population averages (Fowler et al., 2014). This might reflect preferential cleavage by the fission yeast Spo11 ortholog, Rec12, of DNA adjacent to or leaving a nucleosome (Fowler et al., 2014).

### 3.2. Meiotic chromosome architecture

Higher-order chromosome architecture plays an important role in governing DSB hotspot localization. Meiotic chromosomes are characterized by replicated sister chromatids organized into linear arrays of chromatin loops that emanate from a central chromosome axis (Figure 1C) (Blat et al., 2002; Borde and de Massy, 2013). This chromosome organization is dependent on cohesin rings that encircle the sister chromatids (Blat et al., 2002; Borde and de Massy, 2013). DSBs in budding yeast are known to occur primarily within the emanating chromatin loops, while most of the Spo11 accessory proteins that are essential for DSB formation are located on the cohesin-rich axis (Panizza et al., 2011). This is consistent with the “tethered-loop/axis complex” model, which proposes that meiotic recombination occurs at loci within chromatin loops that are tethered to the chromosome axis by recombination-promoting factors (Blat et al., 2002). Further supporting this model, the budding yeast PHD finger domain protein and Set1 complex member Spp1 binds to H3K4me2/3 near gene promoters in chromatin loops and interacts transiently with the axis-bound Spo11 accessory protein Mer2, forming a bridge between potential DSB sites and the recombination initiation machinery (Borde et al., 2009; Acquaviva et al., 2013; Borde and de Massy, 2013; Sommermeyer et al., 2013; Adam et al., 2018).

The meiotic cohesin subunit Rec8 shapes the distribution of Spo11 in budding yeast and is required for normal DSB distribution (Kugou et al., 2009). Spo11 has been observed to initially colocalize with Rec8 at axial cohesion sites and to subsequently associate with chromatin loops during DSB formation (Kugou et al., 2009; Ito et al., 2014). Translocation of Spo11 to chromatin loops is proposed to occur via Spp1-mediated tethering, giving rise to an anti-correlation between DSB formation and cohesin binding at hotspots and axis sites (Borde et al., 2009; Acquaviva et al., 2013; Sommermeyer et al., 2013). For example, lower-than-expected frequencies of budding yeast DSB hotspots are observed in proximity to Rec8 binding sites (Ito et al., 2014). Consistent with this, Spo11 enrichment is strongly diminished at DSB hotspots and increased at axis sites in *spp1* mutants (Sommermeyer et al., 2013). Furthermore, inefficient induction of meiotic DSBs near axis sites in wild-type cells suggests that Spo11 may be inactivated or repressed by axial components (Ito et al., 2014). Preferential DSB formation within gene promoters, coupled with enrichment of cohesin toward and downstream of transcription termination sites in budding yeast (Ito et al., 2014; Sun et al., 2015), illustrates how gene organization may contribute to meiotic DSB hotspot localization (Cooper et al., 2016).

In budding yeast, proteins within the ZMM (Zip, Msh, and Mer) group participate in the assembly of the synaptonemal complex and promote crossing over (Lynn et al., 2007). ZMM proteins are thought to protect dHJ-JMs from disassembly by anti-crossover activity, including that of the RecQ helicase Sgs1 (Jessop et al., 2006; Oh et al., 2007), and are required for the formation of ~85% of crossovers (Lynn et al., 2007). However, more DSBs form globally and at hotspots in ZMM mutants (*zip1, zip3*, and *msh5*) than in wild-type budding yeast cells, indicative of a negative feedback loop in which homolog engagement following DSB formation suppresses Spo11 activity and prevents further breaks (Thacker et al., 2014). These mutants also exhibit increased noncrossover:crossover ratios at selected DSB hotspots (Thacker et al., 2014). Additionally, DSB hotspots with the greatest fold change in Spo11-oligo density in *zip3* are more enriched for the axis proteins Hop1 and Red1, and for the axis-localized Spo11 partner proteins Rec114, Mei4 and Mer2 (Thacker et al., 2014). Thus, while Set1 is important for Spo11 targeting to sites for DSB formation (Borde and de Massy, 2013), the ZMM pathway includes feedback circuitry that controls DSB numbers while promoting crossover recombination (Thacker et al., 2014).

## 4. Meiotic DSB and crossover distributions

Budding yeast DSB and crossover densities are anti-correlated with chromosome size, with smaller chromosomes undergoing more DSBs and crossovers per kilobase than larger chromosomes (Pan et al., 2011). This chromosome-scale control of DSB density is thought to be dictated by a suppressive impact of homolog engagement upon the formation of further DSBs (Thacker et al., 2014), as it has been suggested that smaller chromosomes may engage their homologs more slowly on average, thereby extending the period during which breaks can accumulate (Thacker et al., 2014; Lam and Keeney, 2015). In view of the strong broad-scale correlation between DSB and crossover distributions in budding yeast, regulation of DSB density has been proposed to account for much of the variation in crossover density (Pan et al., 2011). Similarly, mouse chromosome size is negatively correlated with crossover density and, to a lesser extent, DSB density (Lange et al., 2016). The steeper slope observed for the relationship with crossover density suggests that chromosome size is a more important determinant of crossover density than regulation of DSB numbers in mouse (Lange et al., 2016).

Despite positive relationships between genome-wide DSB and crossover distributions, fine-scale correlations at *Arabidopsis* crossover hotspots are weaker and variable (Choi et al., 2018). Interhomolog sequence divergence near DSB hotspots may contribute to this discrepancy by inhibiting crossover formation in hybrids between diverged strains used to map crossovers (Choi et al., 2018). In mouse, for example, crossovers are repressed near indels within the *A3* crossover hotspot (Cole et al., 2010). The absence of strong correlations between DSB levels and crossover frequencies at fine scale may also reflect the fact that a minority of DSBs mature into crossovers in plants and mice (3–10%; De Muyt et al., 2009; Cole et al., 2010). Most strand invasion recombination intermediates are resolved as non-crossovers via processes such as SDSA, dissolution of dHJ-JMs, unidirectional endonuclease cleavage of dHJ-JMs, or intersister repair (De Muyt et al., 2012; Hunter, 2015). For example, high-resolution mapping of meiotic recombination initiation sites in maize identified RAD51 ChIP-seq hotspots in all chromosome regions, whereas crossovers are largely confined to sub-telomeric gene-rich regions comprising open chromatin (Li et al., 2015; Rodgers-Melnick et al., 2015, 2016; He et al., 2017).

Fission yeast recombination landscapes are characterized by crossover invariance, which describes a near-uniform genome-wide distribution of crossovers between homologous chromosomes despite considerable variability in DSB levels (Hyppa and Smith, 2010). This mechanism of crossover control biases DSB repair toward the sister chromatid rather than the homologous chromosome, with intersister repair exceeding interhomolog repair ~3:1 at hotspots (Cromie et al., 2006; Hyppa and Smith, 2010). In DSB-cold regions, by contrast, interhomolog repair is favored (Hyppa and Smith, 2010). While the potential existence of crossover invariance in other eukaryotes remains to be investigated, this type of crossover control may help to explain varying DSB:crossover ratios in diverse species (Fowler et al., 2013).

## 5. PRDM9 and histone H3 lysine 4 trimethylation

The histone-lysine trimethyltransferase PRDM9 dictates the position of the vast majority of DSB hotspots in mouse and primate genomes by conferring a dominant mechanism of DSB spatial regulation (Baudat et al., 2010; Berg et al., 2010; Brick et al., 2012). PRDM9 designates DSB hotspots by trimethylation of H3K4 at loci matching the DNA binding specificity of its zinc finger array (Buard et al., 2009; Grey et al., 2011; Smagulova et al., 2011; Diagouraga et al., 2018). A 12-bp motif matching part of a 36-bp PRDM9^B6^ binding sequence is enriched in DSB hotspots identified by SPO11-oligo mapping and SSDS, and adjacent to PRDM9-dependent H3K4me3 peaks in B6 mice (Brick et al., 2012; Baker et al., 2014, 2015; Lange et al., 2016). Interestingly, SSDS-derived DSB hotspots in *Prdm9*^−/−^ mice occur at H3K4me3-marked gene promoters, sites at which DSBs form rarely in wild-type mice (Brick et al., 2012). This reveals a reversion to a masked, ancestral DSB hotspot designation mechanism analogous to that observed in eukaryotes lacking a PRDM9-like mechanism, including budding yeast, birds, dogs and plants (Cooper et al., 2016). PRDM9 thus diverts DSBs away from functionally conserved genomic elements and toward independent H3K4me3 and H3K36me3 markers deposited via its histone methyltransferase activity (Brick et al., 2012; Diagouraga et al., 2018). According to the “hotspot paradox” hypothesis, rapid loss of PRDM9 recognition sequences through biased gene conversion is predicted to result in the evolutionary erosion of hotspots in primate and mouse genomes (Myers et al., 2010; Cole et al., 2014; Baker et al., 2015). PRDM9 evolves rapidly, however, with the emergence of new allelic variants of its zinc finger motif causing DSB landscapes to be recast and the concomitant designation of new hotspots (Berg et al., 2010; Myers et al., 2010; Brick et al., 2012; Baker et al., 2015; Diagouraga et al., 2018).

Mouse SPO11-oligo-derived DSB hotspot midpoints are depleted of H3K4me3 and H3K36me3, while their flanking regions exhibit a continuum of left–right asymmetric enrichment of these marks, together with secondary SPO11-oligo peaks in adjacent valleys in histone H3 lysine trimethylation signal (Lange et al., 2016; Yamada et al., 2017). *Arabidopsis* DSB hotspots are similarly depleted of H3K4me3 and MNase-seq-derived nucleosome signal (Figures 1A,B) (Choi et al., 2018). This indicates that SPO11 preferentially forms DSBs between nucleosomes in mammalian and plant genomes, similar to its ortholog in budding yeast (Pan et al., 2011). Despite this, DSB formation is severely impaired in the absence of the H3K4 methyltransferase Set1 or the Set1 complex member Spp1, or following mutation of the H3K4 residue targeted by the Set1 complex in budding yeast (Borde et al., 2009; Acquaviva et al., 2013; Sommermeyer et al., 2013). Loci that exhibit the greatest reduction in DSB frequency in *set1* mutants are also located within regions marked by high wild-type levels of H3K4me3 deposition (Borde et al., 2009). As discussed, H3K4me3 plays a role in tethering chromatin loops to the chromosome axis for DSB formation and recombination in nearby promoter NDRs (Borde et al., 2009; Acquaviva et al., 2013; Sommermeyer et al., 2013). Furthermore, SPO11-oligo frequency at mouse DSB hotspots is correlated with H3K4me3 signal in flanking regions (*R*^2^ = 0.40) (Lange et al., 2016). In *Arabidopsis*, by contrast, SPO11-1-oligo enrichment in gene promoters is uncorrelated with levels of H3K4me3 on the first nucleosome immediately downstream of gene transcriptional start sites (i.e., when genes are ordered by decreasing SPO11-1-oligo enrichment in gene promoters, there is no apparent relationship with the degree of H3K4me3 enrichment at the +1 nucleosome) (Choi et al., 2018). Similarly, a minority of maize DSB hotspots overlap H3K4me3 sites (He et al., 2017), and budding yeast DSB frequencies are not correlated with H3K4me3 signal (Tischfield and Keeney, 2012), MNase accessibility or transcriptional activity at hotspots (Zhu and Keeney, 2015). Taken together, these findings indicate that while H3K4me3 deposition is a key determinant of DSB frequency in some eukaryotes, additional factors are important for the local control of DSB numbers.

## 6. The hotspot paradox

The “hotspot paradox” predicts that recombination hotspots will be rapidly eliminated from populations in situations where there are strong *cis*-acting sequence determinants of hotspot activity (Boulton et al., 1997). Under this hypothesis, hotspot-activating alleles are rapidly replaced by hotspot-inactivating mutations via biased gene conversion, whereby DSB repair at an active hotspot allele uses an unbroken homolog bearing an inactive allele, conferring a transmission advantage to the recombination-suppressing allele (Úbeda and Wilkins, 2011). This is expected to give rise to dynamic genome-wide DSB landscapes, within which PRDM9-designated hotspots exist transiently in evolutionary time (Lam and Keeney, 2015). This prediction is reinforced by empirical studies and simulations of recombination hotspot activity and evolution in primates and mice (Pineda-Krch and Redfield, 2005; Coop and Myers, 2007; Friberg and Rice, 2008; Úbeda and Wilkins, 2011).

Conversely, the occurrence of DSB hotspots in the promoter NDRs of several other eukaryotes supports an alternative hypothesis, which proposes that hotspots can persist if DSBs form preferentially within genomic features that are conserved over extended evolutionary periods and whose functions and chromatin state are unrelated to their hotspot status (Lam and Keeney, 2015). This is supported by the strong conservation of DSB hotspot positions and intensities among *Saccharomyces* species (Lam and Keeney, 2015) and among *Schizosaccharomyces* species (Zanders et al., 2014), as well as by evolutionarily stable recombination hotspots in birds (Singhal et al., 2015). Many properties of chromatin structure, at both fine and broad scales, are likely constrained due to their functions in essential processes, including transcription, DNA replication, sister chromatid cohesion, chromatin compaction and chromosome segregation (Pan et al., 2011). As chromatin architecture shapes genome-wide DSB distributions, conservation of the DSB landscape is likely to be a common corollary of selective pressures on chromatin structures to maintain functions independent of meiotic recombination (Lam and Keeney, 2015).

## 7. Recombination initiation in repetitive sequences

DSB formation within or adjacent to repetitive elements can lead to homologous recombination between non-allelic repeats, potentially resulting in harmful chromosomal rearrangements and copy-number instability in the germline (Yamada et al., 2017). DSBs are generally suppressed in budding yeast Ty elements, which may reflect a mechanism to preserve genome stability, although elevated DSB levels are associated with some Ty insertions (Pan et al., 2011; Sasaki et al., 2013). This overall trend is consistent with DSB and crossover repression and elevated transposon density within *Arabidopsis* pericentromeric heterochromatin (Choi et al., 2018). Loss of CG DNA methylation in *Arabidopsis met1* mutants causes increased SPO11-1-oligo levels in EnSpm/CACTA and Gypsy elements and within pericentromeres generally, together with loss of pericentromeric nucleosome occupancy (Choi et al., 2018). Comparable impairment of DNA methyltransferase activity in mouse *Dnmt3L*^−/−^ mutants also results in increased SPO11-dependent DSBs in retrotransposons (Zamudio et al., 2015). This is consistent with findings from epigenetic manipulations in *Arabidopsis* showing that RNA-directed DNA methylation (RdDM) targeted to meiotic hotspots suppresses crossover recombination (Yelina et al., 2015). Furthermore, the histone deacetylase Sir2 inhibits meiotic DSB formation and recombination in the repetitive ribosomal DNA (rDNA) array in budding yeast (Gottlieb and Esposito, 1989; Mieczkowski et al., 2007), indicating that DSB suppression by heterochromatin assembly on repetitive DNA is a conserved strategy to safeguard against genome destabilization.

Despite SPO11-1-oligo depletion in *Arabidopsis* pericentromeric regions, however, significant overlap was observed between DSB hotspots and transposable elements generally (Choi et al., 2018). Specifically, *Arabidopsis* DSB hotspots overlap DNA transposable elements within the Helitron, Pogo/Tc1/Mariner and MuDR families more than expected, whereas hotspots overlap DNA elements in the EnSpm/CACTA class and RNA elements in the Gypsy LTR (long terminal repeat), Copia LTR and LINE-1 classes less than expected (Choi et al., 2018). Helitron and Pogo/Tc1/Mariner transposition occurs preferentially in AT-rich gene regulatory sequences, at which nucleosome exclusion is thought to contribute to increased DSB frequencies (Figures 1A,B) (Kapitonov and Jurka, 2001; Guermonprez et al., 2008; Choi et al., 2018). Similarly, although SPO11-oligos are generally underrepresented in mouse repeats (including LINE-1 retrotransposons), elevated SPO11-oligo levels and functional PRDM9 binding sites were observed within DNA elements in the MULE-MuDR, TcMar-Mariner, hAT-Charlie and PiggyBac families (Yamada et al., 2017). By contrast, most maize DSB hotspots are located in repetitive sequences, although DSBs avoid heterochromatin, forming in transposon NDRs and exhibiting DNA hypomethylation (He et al., 2017). These DSB hotspots occur predominantly in Gypsy LTR retrotransposons, which are abundant in the maize genome (He et al., 2017). Similar to *Arabidopsis* and mouse, however, fewer-than-expected maize DSB hotspots occur in Copia LTR and LINE retrotransposons (He et al., 2017).

Citing the hotspot paradox hypothesis, Yamada et al. (2017) speculate that PRDM9 may target some repeat classes for biased gene conversion to inhibit the proliferation of selfish genetic elements. Rapid fixation of hotspot-inactivating mutations would reduce the copy number of PRDM9-targeted transposons in populations (Yamada et al., 2017). As a Krüppel-associated-box (KRAB)-zinc finger protein, PRDM9 may have derived functions to counteract transposon proliferation from an ancestral KRAB factor, many of which have roles in transposon silencing (Wolf et al., 2015; Yamada et al., 2017). Balancing this proposed transposon-antagonizing role of PRDM9 with mechanisms to prevent excessive DSB formation in repeats may be an important contributor to PRDM9 evolution and DSB hotspot designation in mammalian genomes (Yamada et al., 2017). In *Arabidopsis*, significant overlap occurs between comparable classes of DNA elements and DSB hotspots, many of which are located within functionally conserved sequences (Choi et al., 2018). This suggests that the hotspot paradox theory may not be applicable in this case and that *Arabidopsis* hotspots may be more evolutionarily stable. Nonetheless, comparisons between eukaryotes indicate that repeated sequences may influence meiotic recombination initiation landscapes in related ways.

## 8. Beyond hotspots: DSB-dependent spatial regulation

Meiotic DSB hotspots are identified by mapping the DSB landscape in a population of cells. This landscape reveals a continuum of variation within which loci with high probabilities of DSB formation may be detected (Pan et al., 2011). However, spatial regulation that occurs as a consequence of DSB formation is largely obscured within the population average because low proportions of even the most active DSB hotspots are cleaved in individual meiocytes (~10–15%; Cooper et al., 2016). DSB interference, mediated by the DNA damage response (DDR) kinase Tel1^ATM^ in budding yeast, suppresses the formation of clustered DSBs in *cis* over distances of ~70–100 kb (Garcia et al., 2015; Cooper et al., 2016). Loss of Tel1^ATM^ activity allows DSBs to form independently of one another over ±20–100-kb distances, giving rise to DSB formation in neighboring regions at frequencies comparable to those expected by chance. Over distances of ± ~7.5 kb, by contrast, Tel1^ATM^ inactivation permits the formation of adjacent DSBs significantly more frequently than expected, generating localized regions of “negative DSB interference” (Garcia et al., 2015). This short-range effect occurs only between DSB hotspots located within the same chromatin loop domain (Garcia et al., 2015; Cooper et al., 2016). Coincident DSB formation at adjacent intra-loop hotspots in the absence of *cis*-interference suggests that hotspots within the same loop domain are “primed” for cleavage. Cooper et al. (2016) speculate that tethering of a loop to the chromosome axis may pre-activate the loop and the hotspots within, an effect suggested to be concealed by Tel1^ATM^-dependent *cis*-interference, which restricts DSB formation to only one of the primed intra-loop hotspots. Spatial regulation of meiotic DSB formation also occurs in *trans* via a mechanism involving Tel1^ATM^ and Mec1^ATR^, another DDR signal transduction kinase (Zhang et al., 2011). Following DSB formation on a chromatid, *trans*-interference inhibits DSB formation at the corresponding locus on its sister, its homolog or frequently both. This mechanism is thought to ensure that an intact template is available for DSB repair, and to prevent DSB formation at allelic loci on both homologs (Zhang et al., 2011). Meiotic DSB interference along and between chromatids is therefore likely important for ensuring even spacing of recombination events, thereby contributing to stable interhomolog interactions that facilitate proper chromosome pairing and successful completion of meiosis (Zhang et al., 2011; Garcia et al., 2015; Cooper et al., 2016).

## 9. Future prospects

Genome-wide DSB mapping in different eukaryotes has revealed diversity with regard to the hierarchical combinations of factors that shape meiotic recombination landscapes and hotspots. These distinctions highlight the importance of studying DSB landscapes in diverse eukaryotes and beyond model organisms. Efforts to elucidate the mechanisms that determine DSB hotspot designation may inform genetic or epigenetic manipulations intended to reshape naturally constrained meiotic recombination landscapes. For example, the presence of hotspots in conserved genomic elements, such as nucleosome-depleted promoters, has relevance for targeting crossover recombination to specific loci in plants (Sarno et al., 2017). Manipulation of recombination has the potential to generate greater genetic diversity among gametes for accelerated crop improvement. Such approaches should be considered with caution, however, as forced recombination within repetitive heterochromatin also has the potential to compromise genome integrity in the germline.

## Author contributions

AT and IH wrote and edited the manuscript. AT created Table 1 and IH created Figure 1. Both authors approved the work for publication.

### Conflict of interest statement

The authors declare that the research was conducted in the absence of any commercial or financial relationships that could be construed as a potential conflict of interest.
